# Socioeconomic drivers of vaccine uptake: An analysis of the data of a geographically defined cluster randomized cholera vaccine trial in Bangladesh^☆^

**DOI:** 10.1016/j.vaccine.2018.04.084

**Published:** 2018-07-25

**Authors:** Amit Saha, Andrew Hayen, Mohammad Ali, Alexander Rosewell, C. Raina MacIntyre, John D. Clemens, Firdausi Qadri

**Affiliations:** a School of Public Health and Community Medicine, UNSW Australia, NSW, Australia; b International Centre for Diarrhoeal Disease Research Bangladesh (icddr,b), Dhaka, Bangladesh; c Australian Centre for Public and Population Health Research, Faculty of Health, University of Technology Sydney, Australia; d Johns Hopkins Bloomberg School of Public Health, Baltimore, USA; e UCLA Fielding School of Public Health, Los Angeles, USA; f Korea University School of Medicine, Seoul, South Korea

**Keywords:** Oral cholera vaccine, Vaccination program, Vaccine effectiveness, Socio-economic drivers, Vaccine uptake, Geographic information system

## Abstract

**Background:**

Evaluations of oral cholera vaccines (OCVs) have demonstrated their effectiveness in diverse settings. However, low vaccine uptake in some settings reduces the opportunity for prevention. This paper identifies the socioeconomic factors associated with vaccine uptake in a mass vaccination program.

**Methods:**

This was a three-arm (vaccine, vaccine plus behavioral change, and non-intervention) cluster randomized trial conducted in Dhaka, Bangladesh. Socio-demographic and vaccination data were collected from 268,896 participants. A geographical information system (GIS) was used to design and implement the vaccination program. A logistic regression model was used to assess the association between vaccine uptake and socioeconomic characteristics.

**Results:**

The GIS supported the implementation of the vaccination program by identifying ideal locations of vaccination centres for equitable population access, defining catchment areas of daily activities, and providing daily coverage maps during the campaign. Among 188,206 individuals in the intervention arms, 123,686 (66%) received two complete doses, and 64,520 (34%) received one or no doses of the OCV. The vaccine uptake rate was higher in females than males (aOR: 1.80; 95% CI = 1.75–1.84) and in younger (<15 years) than older participants (aOR: 2.19; 95% CI = 2.13–3.26). Individuals living in their own house or having a higher monthly family expenditure were more likely to receive the OCV (aOR: 1.60; 95% CI = 1.50–1.70 and aOR: 1.14; 95% CI = 1.10–1.18 respectively). Individuals using treated water for drinking or using own tap as the source of water were more likely to receive the OCV (aOR: 1.23; 95% CI = 1.17–1.29 and aOR: 1.14; 95% CI = 1.02–1.25 respectively) than their counterpart. Vaccine uptake was also significantly higher in participants residing farther away from health facilities (aOR: 95% 1.80; CI = 1.36–2.37).

**Conclusion:**

The GIS was useful in designing field activities, facilitating vaccine delivery and identifying socioeconomic drivers of vaccine uptake in the urban area of Bangladesh. Addressing these socioeconomic drivers may help improve OCV uptake, thereby effectiveness of the OCV in a community.

## Highlights

• Use of the GIS facilitated designing improved vaccination strategy in a large clinical trial.
• Two-third of the study population received two complete doses of the oral cholera vaccine.
• Vaccine uptake was significantly higher among females and among younger subjects.
• Individuals living farther away from the health facilities were more like to receive the vaccine.
• Socioeconomic drivers of vaccine uptake should be considered in planning for vaccination strategy.

## 1 Introduction

Cholera is a severe diarrheal disease causing 1.3–4.0 million cases and 21,000–143,000 deaths annually [1]. As the organism, *V. cholerae* is a part of the normal flora in the surface water of the earth [2], the disease is not eradicable. Therefore, improvements in socio-economic factors and infrastructure, access to safe drinking water and improvement of sanitation systems, vaccination and standard medical treatment are considered as key interventions towards reducing the burden of cholera [3], [4].

Due to cholera's severity and rapid progression, prevention and early treatment are essential. Recent advancements in surveillance, medical treatment and the availability of affordable cholera vaccines have helped reduce the case fatality ratio over time [5]. In the last ten years, oral cholera vaccines (OCVs) have been deployed in both mass vaccination programs, feasibility studies and during outbreaks [6], [7], [8], [9]. However, vaccine uptake rates have varied in different geographic settings [10]. The uptake rate of two doses of OCV was 74% in Vietnam, 76% in Uganda refugee camp and 76% in Guinea during outbreak situations [11], [12], [13]. When used in mass vaccination programs, the vaccine uptake rate was 79% in rural Haiti [14], but much lower in Mozambique (41%), Zanzibar (50%) and India (60%) [9], [15], [16].

OCVs have been playing an important role in prevention and control efforts [17]. It is known that vaccine effectiveness in the community depends on the rate of vaccine uptake in the population [18]. As OCVs have been shown to provide direct and indirect protection [19], higher levels of vaccine coverage in the community lower the risk of cholera among residents in the community [19], [20] meaning the broader community will benefit because of herd immunity from the vaccine [19].

In early 2011, a cholera vaccine effectiveness study was conducted in Bangladesh targeting 268,869 people in a low income, urban area of Dhaka using a geographically defined cluster randomized trial [21]. Two dose vaccine uptake in the trial was 66% [6]. Following recent successful adoption in large-scale vaccine trials, we used a geographic information system (GIS) to support trial implementation [22].

In this study, we describe the use of GIS in a large field trial of a cholera vaccine. We also investigated the socioeconomic drivers of vaccine uptake in this clinical trial.

## 2 Method

### 2.1 The study area

The study was conducted in Mirpur in Dhaka, Bangladesh, which is one of the most densely populated cities in the world [23]. Surveillance data from icddr,b (International Centre for Diarrhoeal Disease Research, Bangladesh) hospitals shown that 6 of the 16 wards (the lowest level administrative unit) in Mirpur had a high incidence of cholera cases (2–6 cases per 1000 diarrheal hospitalizations). These wards were selected for conducting the study [6]. The population in the study was registered through a census survey conducted by trial staff.

### 2.2 Constructing the GIS database

**Fig. 1:**
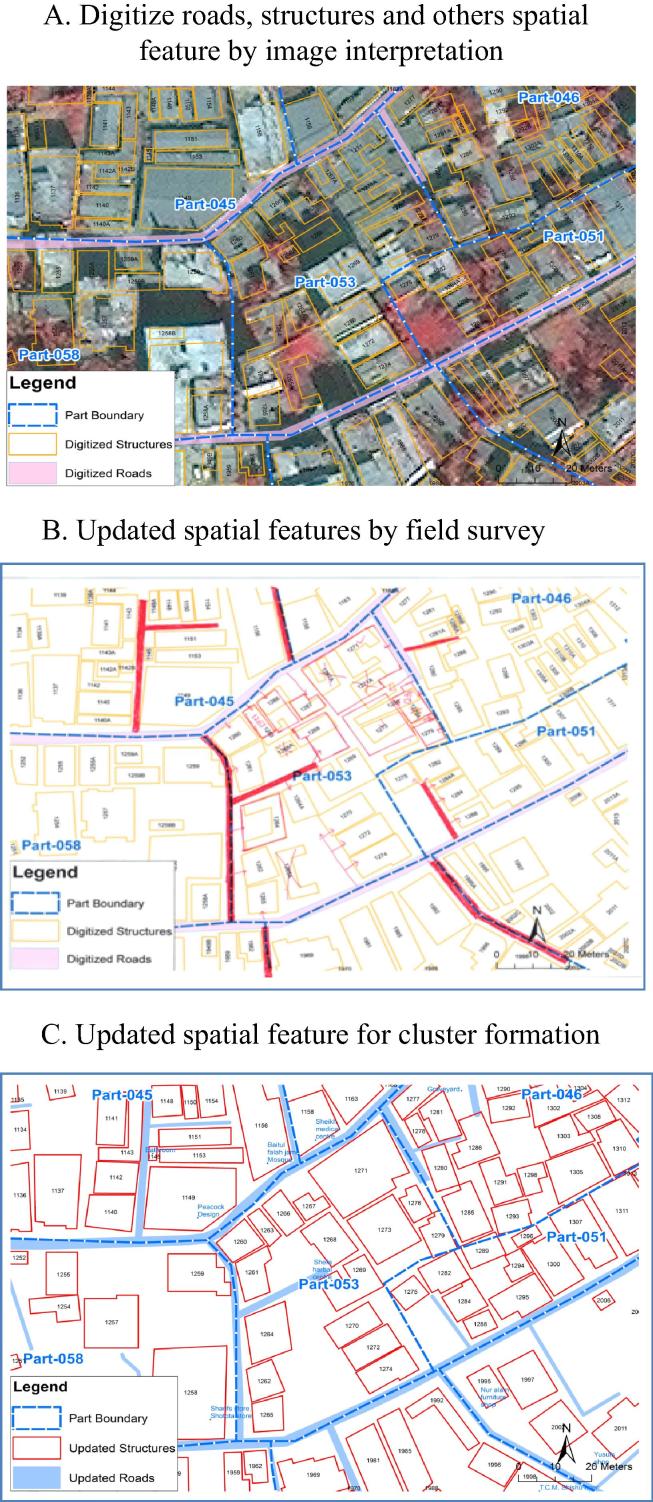
Creation of the spatial database for the study area.

A geographic information system (GIS) was used to monitor vaccine uptake on a daily basis during the campaign and as a research tool. The satellite derived Quick Bird image constructed the household level GIS. The image was enhanced using an image processing software package (ERDAS Imagine, Atlanta, USA) to facilitate the digitization of house parcel boundaries. Differential GPS (Global positioning system) was used to capture data at several identifiable points on the image to be used as ground control points (GCPs). Most of the GCPs were selected from the periphery of the study area so that possible errors would converge towards the middle of the area. The GPS data were collected in the WGS-84 (World Geodetic Systems-84) datum in the latitude/longitude system and were subsequently transformed into the Everest 1830 (Bangladesh Transverse Mercator, BTM). The GCP coordinates within the BTM projection were then integrated with the satellite images using the ERDAS Imagine software for geo-referencing. The resultant root mean square (RMS) errors were approximately two meters, which was considered sufficiently accurate for the purpose of constructing the GIS database. After geo-referencing the image, on screen digitization was performed keeping the image as a backdrop (Fig. 1). Each geographic feature such as structures, roads, water bodies was stored as a separate entity in the GIS database. A ground survey was done with the digitized map to link the structures to the household data. Subsequently, the GIS database was routinely updated with the demographic surveillance system during the period of study.

### 2.3 Cluster formation and randomization

**Fig. 2:**
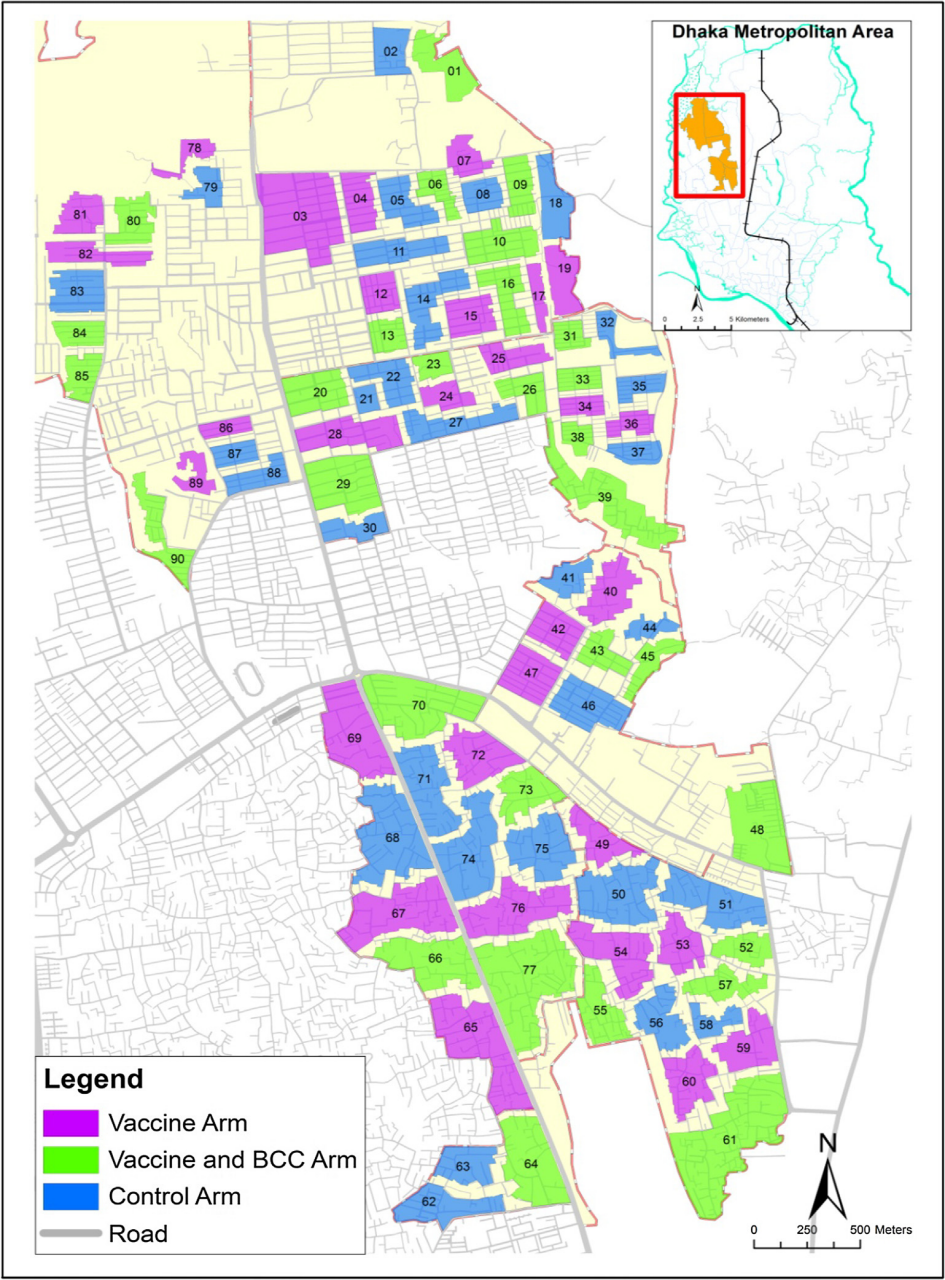
The geographic clusters on the study. The cluster numbers are shown inside the clusters. The inset shows the geographic position of the study area in the Dhaka Metropolitan area.

There were three arms of the study: (i) vaccine (ii) vaccine plus behavior change component (BCC) and (iii) non-intervention, and the design included 30 clusters in each arm of the study. Based on the population size, we estimated an average of 3000 individuals would be in each cluster to balance the trial population across the clusters. To avoid any spillover of the effect of intervention in nearby clusters, we set buffer of at least 30 m between the two neighboring clusters. After creating the clusters, they were randomly assigned into the three arms of the study (Fig. 2).

### 2.4 Vaccination and disease surveillance

Of the 268,896 individuals living in the 90 clusters, 95,115 were in the vaccine arm; 93,091 in the vaccine plus behavior change arm; and 80,690 in non-intervention arm. The OCVs were distributed in the vaccine and the vaccine plus behavior change arms comprising 60 clusters that included 188,206 individuals.

Two doses of OCV ‘Shanchol’ containing inactivated *Vibrio cholerae* O1 and *V. cholerae* O139 bacteria were used in the study [24]. Individuals who were aged one year or older and who were not pregnant were invited to be immunized, and after obtaining informed consent, the two doses of the OCV were offered at an interval of two weeks [6], [25].

**Fig. 3:**
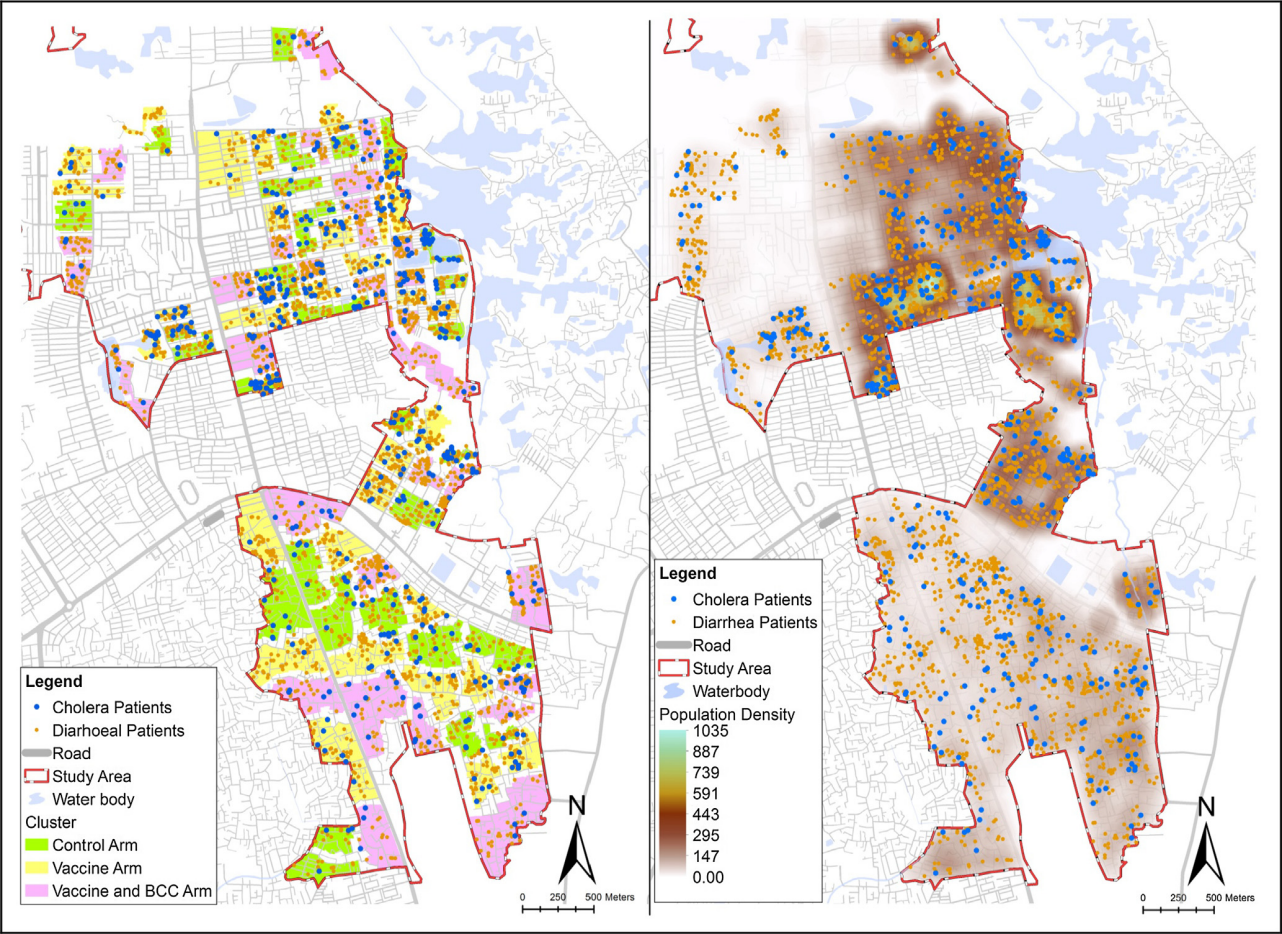
Spatial distribution of diarrhoea and cholera cases with population density.

Following vaccination, hospital based passive surveillance was carried out in 12 health facilities. Diarrheal stools were collected from all patients presenting to the 12 health facilities and samples were tested to identify cholera and Enterotoxigenic *Escherichia coli* (ETEC) pathogens [6]. All these data were aggregated with the GIS data set for analysis and clusters drawn where the diarrhoea and cholera cases were identified (Fig. 3).

### 2.5 Funding source and ethics

The project was funded by the Bill and Melinda Gates Foundation (Grant No. OPP 50419). The protocol was approved by the Government of Bangladesh; the IRB of icddr,b and institutional review board of the International Vaccine Institute (IVI), Seoul, Korea. The study was also monitored by the data and safety monitoring board of icddr,b (DSMB). Verbal informed consent was obtained from an adult in each household during population enumeration and written informed consent was obtained from all adult participants and legal guardians for children before vaccination. The protocol was registered at ClinicalTrials.gov number, NCT01339845.

### 2.6 Statistical analysis

Our primary outcome was receipt of two doses of the OCV. Baseline socioeconomic characteristics were collected from all participants. Potential socioeconomic factors associating with vaccine uptake included in the analysis were: age (<15 years versus. ≥15 years), sex (male vs. female), size of household (≤4 members vs. 5 members or more), household type (owned by the occupants vs. rented), construction material of the floor of house (concrete vs. others), education level of the household head; the household head was recognized by other members of the household (formal education vs. no formal education), type of toilet use (sanitary vs. non-sanitary), treatment of drinking water (treated with tablet, chemical or filter vs. non-treated), source of drinking water (own tap or tube-well vs. common tap or tube well), reported diarrhoea in 48 h prior to registration in the project’s census survey (yes vs. no), and distance (km) to the nearest health facility.

We used a logistic regression model to assess the association of vaccine uptake with socioeconomic characteristics. In the model, we fitted a random intercept to account for clustering of individuals within the household and a fixed effect for trial cluster. We were unable to fit random intercepts for household and cluster in the model because of large sample size in the study. We chose to model household using random intercepts rather than trial clusters because preliminary analyses indicated a very strong intracluster correlation by household. We also note that because of collinearity, we were unable to include both trial arm and cluster in the model.

All categorical and continuous variables were included in the univariate analysis and only those variables were selected for the multivariable model which were associated with the outcome at p < 0.2. The backward elimination process was chosen in the multivariable model to eliminate variables that were associated with the outcome at p > 0.05. Analyses were conducted using SAS version 9.4 [25].

### 2.7 Role of the funding source

The funder of the study had no role in data collection, data analysis, data interpretation, or writing of the manuscript. The corresponding author had full access to all data in the study and had final responsibility for the decision to submit for publication.

## 3 Results

### 3.1 Monitoring the vaccination program

**Fig. 4:**
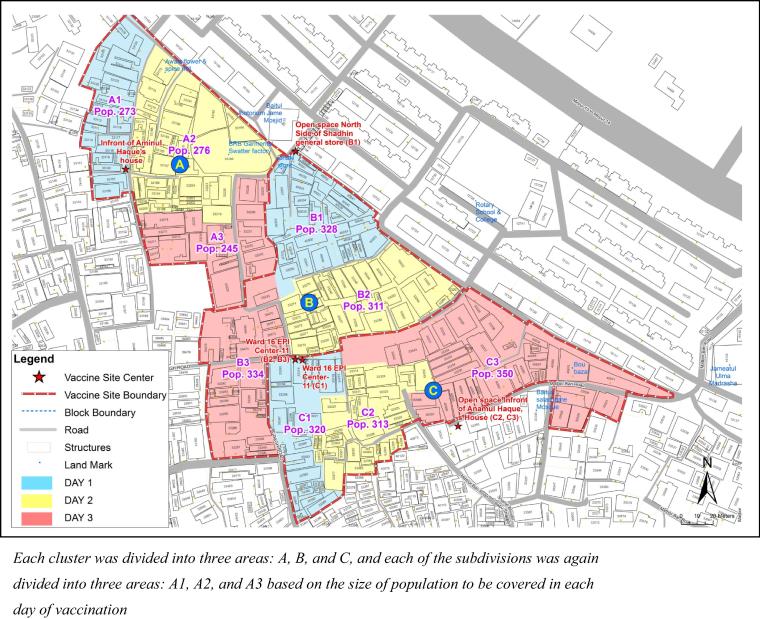
A sample of the part map for vaccine operation.

A GIS-based map was produced for all vaccination sites, their catchment area, and the target population. The map was divided into several parts (called ‘part’ maps) based on each day of vaccine delivery. Approximately 3000 individuals were covered in three days of vaccination in an area using three vaccination sites (∼333 individuals in a site) (Fig. 4). The vaccination sites were established in a way that were easily accessible for all people in the cluster and in a known location used by the national immunization program. The study staff from the study area used the part map for mobilizing the target population in the cluster to come to the vaccination centre. At the end of each day, a meeting was held to review the progress of vaccination activity for the day. At the end of day 3, the list of missed participants was generated and new coverage maps were prepared to assist the volunteers to identify people in the cluster who were unvaccinated during their scheduled date of vaccination and to motivate them to take the vaccine on the next day of the campaign.

### 3.2 Socioeconomic drivers of vaccine uptake

Among 188,206 individuals in the intervention arms, 123,686 received two complete doses of the OCV (vaccinated) and 64,520 received one or no dose of the vaccine (non-vaccinated), yielding 66% coverage of complete dose recipients. There were 46,153 households included in the study, with an average of 4 members in each household. Vaccine uptake was significantly higher in females than males (Table 1). Females had 80% higher odds of receiving two doses of the vaccine than males (adjusted odds ratio aOR: 1.80; 95% CI = 1.75–1.84) Younger participants (1 to <15 years) had a significantly higher uptake of vaccine than older participants (aOR: 2.19; 95% CI = 2.13–3.26).

**Table 1:** Socio-economic predictors of oral cholera vaccine uptake in a randomized controlled vaccine trial in Bangladesh (n = 188,206), mixed models analysis.

Characteristics	Two doses recipients (n = 123,686)	Single or no dose recipients (n = 64,520)	Random household and fixed cluster effects			
			cOR (95% CI)	P-value	aOR (95% CI)	P-value
Age – no (%)						
<15 yrs	42,704 (74.8)	14,366 (25.2)	2.14 (2.08–2.20)	<0.0001	2.19 (2.13–2.26)	<0.0001
≥15 yrs	80,982 (61.8)	50,154 (38.2)	Ref.		Ref.	

Sex – no (%)						
Female	67,471 (69.6)	29,402 (30.4)	1.81 (1.76–1.85)	<0.0001	1.80 (1.75–1.84)	<0.001
Male	56,215 (61.5)	35,118 (38.5)	Ref.		Ref.	

Household members – no (%)						
1–4 members	48,160 (62.0)	29,514 (38.0)	0.68 (0.65–0.70)	<0.0001	0.77 (0.74–0.80)	<0.001
>4 members	75,526 (68.3)	35,006 (31.7)	Ref.		Ref.	

Household type – no (%)						
Own household	28,014 (72.0)	10,881 (28.0)	1.68 (1.59–1.78)	<0.0001	1.60 (1.50–1.70)	<0.001
Rented and Supplied by employer	95,672 (64.0)	53,639 (36.0)	Ref.		Ref.	

Education – no (%)						
No formal education	53,613 (66.9)	26,537 (33.1)	0.88 (0.85–0.92)	<.0001	0.89 (0.85–0–0.93)	<0.001
Formal education ≥ class 1	70,063 (64.9)	37,963 (35.1)	Ref.		Ref.	

Floor of the household – no (%)						
Concrete	111,034 (65.7)	57,929 (34.3)	0.96 (0.88–1.03)	0.267	–	–
Mud/wood/bamboo/others	12,313 (65.5)	6499 (34.6)	Ref.			

Toilet used – no (%)						
Sanitary with flush	723 (62.5)	434 (37.5)	0.86 (0.66–1.12)	0.290	–	–
Sanitary without flush	122,963 (65.7)	64,086 (34.3)	Ref.			

Source of drinking water – no (%)						
Own Tap/Well/Hand pump	6821 (71.0)	2780 (29.0)	1.32 (1.20–1.47)	<0.0001	1.14 (1.02–1.25)	0.016
Communal and shared Tap/Well/Hand pump	116,865 (65.4)	61,740 (34.6)	Ref.		Ref.	

Type of drinking water – no (%)						
Treated (Boiled/filtered/chemical)	68,157 (66.5)	34,364 (33.5)	1.19 (1.14–1.25)	<0.0001	1.23 (1.17–1.29)	<0.001
Not treated	55,529 (64.8)	30,156 (35.2)	Ref.		Ref.	

Diarrhoea within 48 h at the time of baseline survey – no (%)						
Yes	1650 (69.7)	719 (30.4)	1.28 (1.13–1.44)	<0.0001	–	–
No	122,036 (65.7)	63,799 (34.3)	Ref.			

Total monthly family expenditure						
>USD 75	115,504 (66.0)	59,537 (34.0)	1.17 (1.13–1.22)	<0.0001	1.14 (1.10–1.18)	0.0007
<USD 75	7699 (62.2)	4672 (37.8)	Ref.		Ref.	

Distance of the hospital (km, SD)	0.58 ± 0.3	0.57 ± 0.3	1.83 (1.39–2.40)	<0.0001	1.80 (1.36–2.37)	<0.001

^*^Plus-minus values are means ± SD.

*P* < 0.05; statistically significant.

cOR: Crude odds ratio, aOR adjusted odds ratio.

Missing variables values: education: 30 values, Floors: 431 values, Baseline diarrhoea: 2 values, Family expenditure: 794 values.

Individuals who owned their house and living in a household having higher monthly family expenditure were more likely to receive two doses of the OCV compared to individuals who resided in rental housing or having lower monthly family expenditure (aOR:1.60; 95% CI = 1.50–1.70 and aOR:1.14; 95% CI = 1.10–1.18 respectively). Individuals whose household head had formal education had a lower vaccine uptake than those whose household head had no formal education (aOR: 0.89; 95% CI = 0.85–0.93). It was also observed that individuals who used their own tap and those who used treated water for drinking were more likely to receive the OCV compared with individuals who use a communal tap and do not treat water for drinking (aOR:1.14; 95% CI = 1.02–1.25 and aOR:1.23; 95% CI 1.17–1.29, respectively). Vaccine uptake was significantly higher among individuals residing farther away from health facilities (aOR: 1.80; 95% CI = 1.36–2.37) than those who live close to a health facility.

The intracluster correlation coefficient in the final model was 0.46, indicating a high level of clustering at the household level.

## 4 Discussion

This study highlights the usefulness of GIS in generating information on spatial distribution of households for vaccination site selection and outreach teams to enable easy access and communication with the study population. Having accurate location data of all households in a cluster allowed preparation of maps to plan for social mobilization efforts, which allowed study personnel to easily locate and visit the households and invite its members for vaccination. The daily coverage maps were useful in identifying the locations of unvaccinated populations in real time, and the community mobilizers visited those locations to encourage people to come to the vaccination centre on next day. Note that such a strategy was used to eradicate polio in the Democratic Republic of Congo, where Google Earth was used to support an intensive vaccination and treatment campaign [26]. A study demonstrated that GIS can support rapid, targeted vaccination efforts by deploying vaccination teams to highly affected areas and to identify suitable locations for storing and administering vaccines [27]. A similar application of GIS based mapping has also been used in treatment programs for the diseases such as Gonorrhea, HIV/AIDS and hepatitis C [28]. One of the important uses of GIS is to identify areas with high risk for the disease [29]. Identifying such areas of increased risk and the drivers of the risk would help better planning for an intervention program in controlling diseases.

This study suggests that socioeconomic drivers need to be taken in consideration to improve vaccine uptake during vaccine trials. We observed that women were more likely to be vaccinated than men, consistent with previous studies [12]. An important finding from the study was the lower vaccine coverage among adults. Adult males are typically the key income generating member in families in Bangladesh [30], and it is possible that they were unable to come to the vaccination sites during working hour. Offering vaccination outside working hours may be an option to increase vaccine uptake among adults, particularly men as well as in sites frequented by men.

Individuals who lived in their own home or living in a household having higher monthly household expenditure (considered a proxy for wealth) had higher vaccine uptake, suggesting that vaccine uptake is associated with household economic conditions, as has been observed in other studies [31]. Education is an important factor in vaccine uptake [32]. In this study we observed that vaccine uptake was higher in people who lived in a household where the household head had no formal education, although the effect was not strong (66.9% versus 64.9%).

Vaccine uptake was also influenced by the distance to health facilities, i.e., those who lived farther from the hospital were more likely to receive the vaccine. Health system access is multi-faceted, and includes drivers such as distance to the health facility, social barriers, economic conditions as well as health-seeking behavior of the community [33]. People who lived at a greater distance from health facilities might have considered the vaccination program as an opportunity to protect themselves against cholera, as accessibility to healthcare is potentially more difficult for them. Future vaccination trials in Bangladesh could consider community based clinics to overcome this issue.

A strength of our study was that the data were obtained from a very large clinical trial, and the study was conducted in real field conditions. The trial was the largest cholera vaccine trial and we used GIS for planning and implementation of the trial and analysis of the trial data. Future clinical trials and immunization programs would benefit from using the GIS technology. The main limitation of this study is that the mass vaccination campaign was conducted in a highly mobile population. Therefore, reaching all the target population in the existing database was not possible since many individuals moved to another area between registration and vaccination. The other limitation is that the trial was designed to evaluate the effectiveness of the OCV, which means the data came from a study carried out under ideal field condition, thus the results of the study may not be generalizable to real field settings.

## 5 Conclusion

The GIS was found very useful in defining the catchment population for daily vaccination activities and enabling vaccine delivery in order for improving performance of vaccine uptake in the trial. This study suggests that socioeconomic drivers of vaccine uptake need to be addressed in a mass vaccination campaign. Individual’s health and hygiene behavior as well as health seeking behavior may also regulate the decision for vaccine uptake in developing countries. In conclusion, the GIS facilitated vaccination strategy in our study, and addressing of the socioeconomic drivers of vaccine uptake, as observed in our study, may increase and spatially equitable performance of vaccine uptake in a mass vaccination campaign in developing countries.
